# Comparison of ^68^Ga-FAPI and ^18^F-DOPA PET/CT in patients with medullary thyroid cancer

**DOI:** 10.3389/fendo.2026.1824054

**Published:** 2026-06-24

**Authors:** Daniel A. Hescheler, Dogan Atiyat, Kerstin Lorenz, Alexander Heinzel, Philipp Reschke, Frankis G. Almaguel, Manuela Petersen, Elisabeth Eppard, Joanna Wybranska, Jan Wuestemann, Michael C. Kreissl

**Affiliations:** 1 University Hospital Magdeburg, Division of Nuclear Medicine, Department of Radiology and Nuclear Medicine, Magdeburg, Germany; 2 Martin Luther University of Halle Wittenberg Faculty of Medicine, Department of Nuclear Medicine, Halle (Saale), Germany; 3 Martin Luther University Halle-Wittenberg, Halle (Saale), Department of General, Visceral and Vascular Surgery, Halle (Saale), Germany; 4 Martin Luther University Halle-Wittenberg, Halle (Saale), Department of Nuclear Medicine, Halle (Saale), Germany; 5 University Hospital Frankfurt, Department of Radiology, Frankfurt, Germany; 6 Loma Linda University Medical Center, Department of Radiology, Loma Linda, CA, United States; 7 Clinic for General, Visceral, Vascular, and Transplant Surgery, Magdeburg University Hospital, Magdeburg, Germany

**Keywords:** DOPA, FAPI, medullary thyroid cancer, PET, recurrence

## Abstract

**Introduction:**

Accurate staging is paramount for the effective management of medullary thyroid carcinoma (MTC). Imaging cancer-associated fibroblasts utilizing Gallium-68-labeled fibroblast activation protein inhibitors (FAPI) presents a novel approach for molecular imaging across various cancers, including MTC. This study aimed to compare the diagnostic accuracy of FAP-targeted imaging (^68^Ga-RTX-1363S, abbreviated as ^68^Ga-FAPI) with the established imaging standard, ^18^F-DOPA, for the detection of metastases in patients with MTC.

**Methods:**

This retrospective study compared ^18^F-DOPA- and ^68^Ga-FAPI PET/CT imaging using per-patient and per-lesion analyses in patients with recurrent MTC. Imaging findings were correlated with morphological imaging (CT/MRI/sonography) or histopathology as gold standard. Quantitative assessment included a comparison of standardized uptake values (SUVs) and tumor-to-background ratios (TBRs).

**Results:**

Nine patients (mean age 55 years, range 34–80 years) with 62 lesions were included. Compared to ^18^F-DOPA, ^68^Ga-FAPI showed significantly higher lesion detection rates for lymph node metastases (true-positive [TP] rate: 100% [8/8] vs. 50% [4/8]; TBR: 10.9 vs. 6.9), lung metastases (TP rate: 85.7% [12/14] vs. 42.9% [6/14]; TBR: 6.1 vs. 3.6), and liver metastases (TP rate: 100% [22/22] vs. 23% [5/22]; TBR: 24.1 vs. 2.7).

**Conclusion:**

In direct comparison, ^68^Ga-FAPI detected a higher number of metastases with higher TBR values. ^68^Ga-FAPI PET/CT holds promise for improving staging in MTC. However, these findings necessitate confirmation in a larger, prospective study.

## Introduction

Medullary thyroid carcinoma (MTC) is a rare malignancy originating from the parafollicular C-cells of the thyroid gland (1, 2). It represents a rather uncommon subtype of thyroid cancer, accounting for only about 4% of all thyroid malignancies (3). MTC occurs in two forms: sporadic (75%) and hereditary (25%), the latter is usually associated with germline mutations in the RET proto-oncogene, leading to multiple endocrine neoplasia type 2 (MEN2) syndromes (4, 5). The diagnostic workup for MTC involves a multimodal approach, including serum calcitonin and carcinoembryonic antigen (CEA) measurements, genetic testing, neck ultrasonography, single-photon emission computed tomography (SPECT), and positron emission tomography (PET) (6). Nuclear imaging techniques offer non-invasive methods for detecting early pathophysiological alterations in MTC (6). Historically, SPECT radiopharmaceuticals, such as Indium-111-labeled somatostatin analogues, were utilized. However, PET radiopharmaceuticals offer superior spatial resolution and have become the predominant modality in the past decade (6). The most frequently employed PET radiopharmaceuticals in this context include fluorine-18-fluorodeoxyglucose (FDG) (6), ^18^F-dihydroxyphenylalanine (^18^F-DOPA) (7), and Gallium-68-labeled somatostatin analogues (^68^Ga-DOTA peptides). Among these, ^18^F-DOPA is frequently considered a standard imaging modality due to its targeting of specific amine uptake mechanisms in C-cells. As a precursor of endogenous catecholamines, ^18^F-DOPA enters cells through L-type amino acid transporters (LAT) and is subsequently metabolized into ^18^F-dopamine by cytosolic aromatic L-amino acid decarboxylase (AADC). Because MTC cells exhibit a marked upregulation of both LAT expression and AADC activity, they demonstrate a characteristically high uptake of ^18^F-DOPA in metastatic and recurrent lesions (8, 9). However, the diagnostic efficacy of ^18^F-DOPA remains subject to notable limitations. False-negative results may arise from small tumor volume, dedifferentiation, or lesions situated within or adjacent to areas of physiological radiopharmaceutical uptake. Conversely, false-positive findings can occur due to inflammatory processes driven by elevated amino acid transport in macrophages (10). Most notably, its clinical utility exhibits diminished sensitivity in patients presenting with low or slowly rising serum calcitonin levels (10).

Consequently, the development of a diagnostic agent with promising detection efficacy and potential therapeutic implications remains crucial for improving MTC management. Recently, a novel radiopharmaceutical targeting fibroblast activation protein (FAPI), labeled with ^68^Ga, has emerged as a significant molecular imaging and potential therapeutic tool across a spectrum of cancer types, taking advantage of the prominent desmoplastic stroma reaction in malignant tumors (11, 12). However, the application of ^68^Ga-FAPI PET/CT in the diagnostic evaluation of medullary thyroid cancer has not been extensively investigated. We hypothesized that targeting the desmoplastic stroma reaction/tumor microenvironment via ^68^Ga-FAPI PET/CT can overcome the metabolic-based limitations of standard ^18^F-DOPA imaging, thereby providing higher lesion-to-background contrast and improved detection rates for both primary and metastatic MTC lesions. This study aimed to compare the detection efficiency of primary and metastatic lesions of MTC between standard diagnostic imaging using ^18^F-dihydroxyphenylalanine ^18^F-DOPA and ^68^Ga-fibroblast activation protein inhibitor/ligand (^68^Ga-FAPI).

## Materials and methods

The retrospective study protocol was approved by the ethics committee of the Otto-von-Guericke-University of Magdeburg (87/25) and was conducted in accordance with the Declaration of Helsinki as revised in 2013. All participating patients provided written informed consent. Patients meeting the following eligibility criteria were included: Histologically confirmed MTC and having undergone both ^18^F-DOPA and ^68^Ga-FAPI PET/CT imaging within a 3-month period. Patients with active infections and other malignancies were excluded.

Three out of 12 patients underwent only ^68^Ga-FAPI PET/CT (without a comparative ^18^F-DOPA scan within the defined timeframe) and were excluded. Therefore, a total of nine patients could be analyzed.

### Radiosynthesis

The radiosynthesis of ^68^Ga-RTX-1363S (^68^Ga-FAPI) was performed under Good Manufacturing Practice (GMP) conditions on a GAIA synthesis module Elysia Raytest (Straubenhardt, Germany). [68Ga]GaCl3 from a GalliaPharm generator (Eckert & Ziegler, Berlin, Germany) was collected and purified on a SCX cartridge. After elution of the [68Ga]GaCl3 with eluent solution (RT-10X-S1, ABX, Radeberg, Germany) into a mixture of 50 µg labeling precursor RTX-1363S, 200 µl EtOH and 3 ml acetate buffer solution (pH = 4.5, RT-10X-V1, ABX, Radeberg, Germany) the reaction was heated to 95 °C for 10 min. The product was trapped and washed with water on a Sep-Pak-C18 light cartridge (Waters, Eschborn, Germany). The product was eluted with 1,5 ml 60% EtOH, formulated with 8,5 ml saline and steril-filtered. The radiochemical yield after sterile filtration was about 75%.

The radiochemical synthesis of [18F]fluoro-L-3,4-dihydroxyphenylalanine (^18^FDOPA) was commercially sourced from IASON GmbH (Graz, Austria).

### PET/CT acquisition

PET/CT imaging was performed using a Biograph mCT 64^®^ scanner (Siemens Healthineers, Erlangen, Germany). Imaging was conducted according to the current procedural guidelines of the European Association of Nuclear Medicine (10). For the ^18^F-DOPA imaging; a median activity of 205 MBq of ^18^F-DOPA (range: 181–305 MBq) was administered intravenously, and PET acquisition commenced after a median uptake time of 60 minutes (range: 54–67 minutes). To minimize non-specific uptake during the FAPI imaging, a cold preload (RTX-1363S) was administered intravenously. Following a 10-minute interval, a median activity of 148 MBq (range: 116–176 MBq) of ^68^Ga-RTX-1359R was injected. PET image acquisition for the FAPI tracer was initiated after a median uptake time of 20 minutes (range: 15–30 minutes). All PET data were acquired from the skull base to the mid-thigh, utilizing six to eight bed positions (3 minutes per position; axial coverage: 216 mm; overlap: 89 mm). Concurrently, low-dose CT (tube current: 50 mA; tube voltage: 120 kV; gantry rotation time: 0.5 s; pitch: 0.8) was performed for attenuation correction and anatomical co-registration.

### Data analysis and processing

To evaluate the diagnostic capabilities of the two radiotracers, both per-patient and per-lesion analyses were performed. Two experienced nuclear medicine physicians (DA & DH; each with over 7 years of experience in PET/CT interpretation) independently reviewed both the ^68^Ga-FAPI and ^18^F-DOPA PET/CT, with discrepancies resolved by consensus.

Qualitative evaluation of the PET images involved visual assessment. For quantitative comparison, three-dimensional volumes of interest (VOIs) were meticulously drawn around lesions exhibiting uptake of ^18^F-DOPA or ^68^Ga-FAPI on transaxial PET images. The maximum standardized uptake values normalized to body weight (SUVmax-bw) were recorded for each lesion site for both tracers. The tumor-to-background ratio (TBR) was calculated by dividing the SUVmax of the metastasis by the SUVmax of the corresponding background tissue.

### Reference criteria for imaging diagnosis

Due to the retrospective nature of the study and the clinical management of recurrent MTC, a composite reference standard was employed to verify metastatic lesions. A finding was defined as a true-positive (TP) based on at least one of the following criteria: 1) Histopathological confirmation from surgical resection or biopsy (where available). 2) Morphological correlation on cross-sectional imaging (contrast-enhanced CT, MRI, or ultrasound) showing size, shape, or enhancement patterns characteristic of malignancy. 3) Strict semi-quantitative criteria for cases with limited morphological correlation (e.g., native low-dose CT), where a TBR > 5 was required to classify a focal uptake as a true-positive lesion. Lesions that did not meet these criteria or disappeared during follow-up without treatment were considered false-positive.

### Statistical analysis

Statistical analysis was performed using SPSS version 30.0.0.0 (IBM Corp., Armonk, NY, USA). For non-normally distributed data, continuous variables are expressed as the mean and interquartile range (IQR). The TBR values of ^18^F-DOPA and ^68^Ga-FAPI were compared using the Wilcoxon signed-rank test, with a p-value of <0.05 considered statistically significant.

## Results

**Figure 1:**
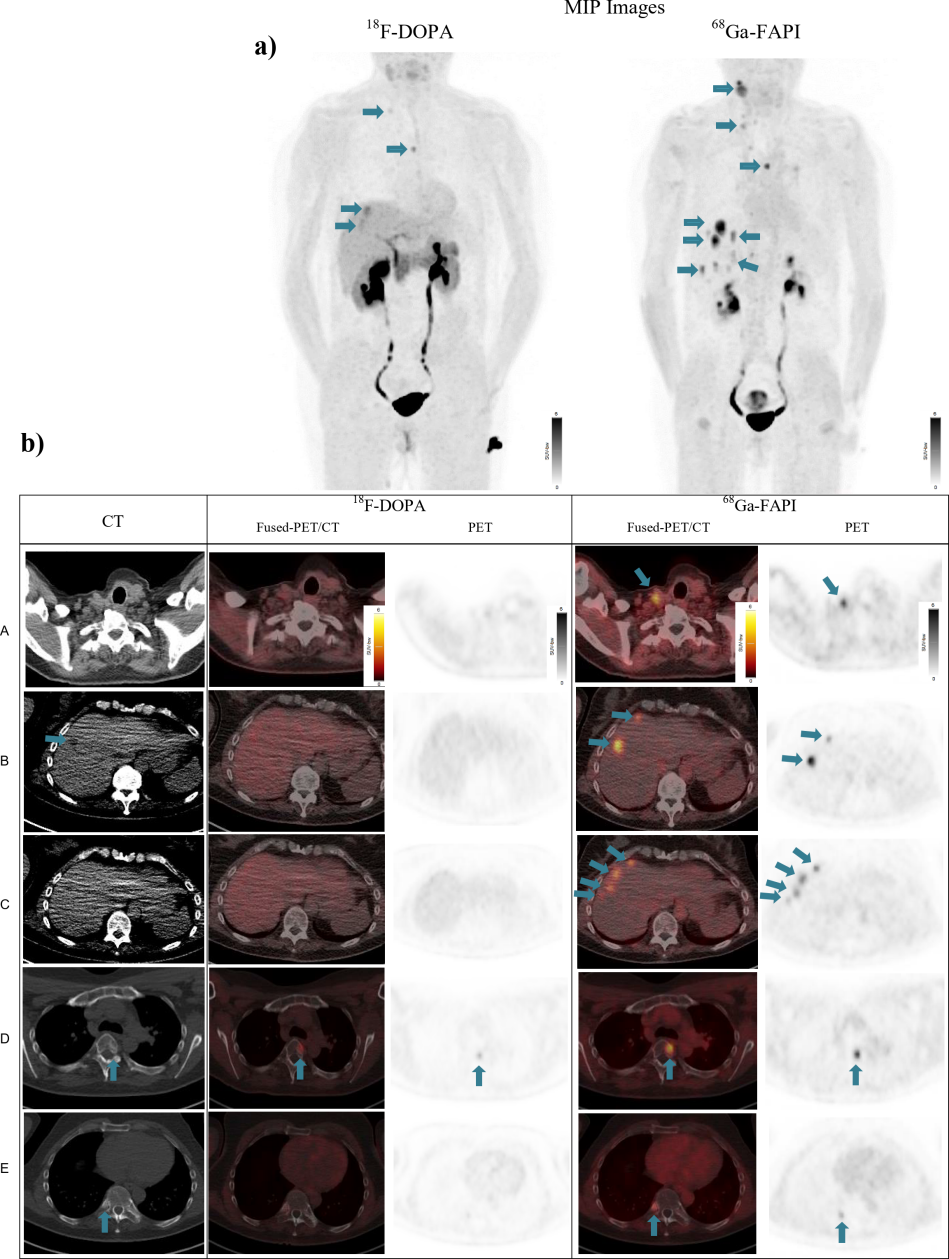
A 53-year-old patient with MTC (pT3 pN1b) underwent thyroidectomy in 2021. Over the subsequent three-year follow-up, the tumor marker initially remained stable before calcitonin levels rose from 4255 pg/ml to 11100 pg/ml. Consequently, ^68^Ga-FAPI and ^18^F-DOPA PET/CT scans were performed within a two-month interval. This figure displays the findings of ^18^F-DOPA and ^68^Ga-FAPI PET/CT, including **(A)** MIP (Maximum Intensity Projection), **(B)** CT images, **(C)** fused PET/CT images, and **(D)** PET images. Row **(A)** illustrates a cervical lymph node metastasis (arrows). Rows **(B, C)** demonstrate liver metastases (arrows) with strong ^68^Ga-FAPI uptake (SUVmax 9.3, TBR = 7.6) versus no discernible ^18^F-DOPA uptake. Row **(D, E)** reveal bone metastases in the T5 vertebral body and the right ninth rib (arrows).

**Figure 2:**
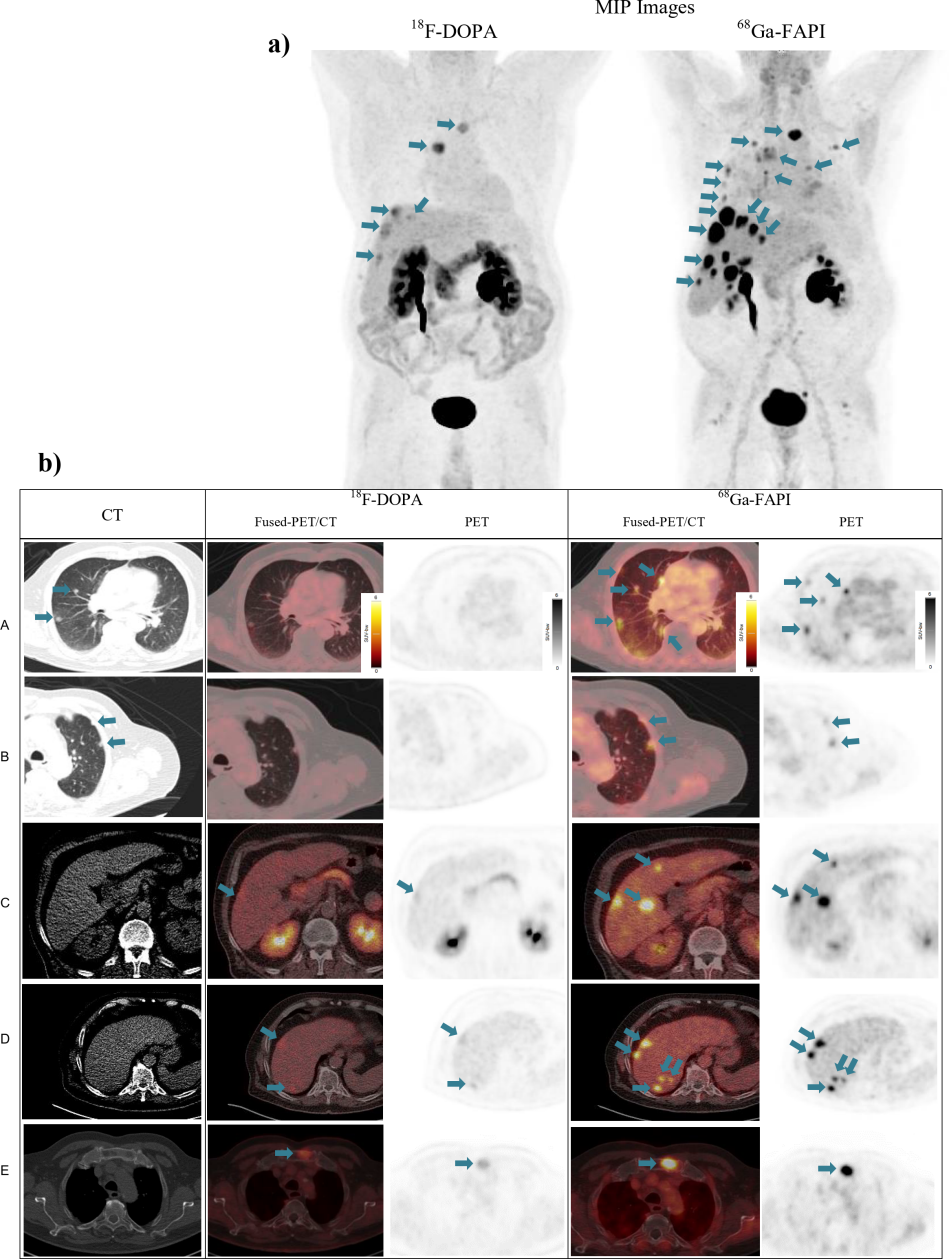
A 68-year-old male patient with sporadic MTC (pT1b) presented with an elevated calcitonin level (158 pg/ml) and subsequently underwent ^18^F-DOPA and ^68^Ga-FAPI PET/CT. This figure displays the resulting images: **(A)** maximum intensity projection (MIP), **(B)** CT, **(C)** fused PET/CT, and **(d)** PET. Rows **(A, B)** reveal multiple lung metastases (arrows) exhibiting strong ^68^Ga-FAPI uptake (SUVmax = 8.4, TBR = 11.3) in contrast to the absence of ^18^F-DOPA uptake. Similarly, Rows **(C, D)** demonstrate multiple liver metastases (arrows) with strong ^68^Ga-FAPI uptake (SUVmax = 9.9; TBR = 3.4) where no ^18^F-DOPA uptake was observed. Row **(E)** shows a bone metastasis in the manubrium of the sternum (arrows).

A total of nine MTC patients (6 men and 3 women) were enrolled in the study, exhibiting a mean age of 55 years (range: 34–80 years). All patients had undergone prior total thyroidectomy and cervical lymph node dissection. Three patients (33%) had a history of surgical resection for recurrence. At the time of imaging, the median serum calcitonin level was 715 pg/mL (interquartile range [IQR]: 232-4210), and the median basal serum CEA level was 8.1 ng/mL (IQR: 5.7-75.0) (**Table 1**). Two case examples from patients with medullary thyroid cancer and elevated tumor markers are presented, showcasing the ^68^Ga-FAPI-PET/CT and ^18^F-DOPA-PET/CT scans (**Figures 1**, **2**).

**Table 1:** Patient characteristics.

Nr	Gender	Age	TNM classification	Tumor markers		^68^Ga-FAPI	LN Mets	Distant mets	^18^F-DOPA	LN mets	Distant mets
				Calcitonin (pg/l)	CEA (µg/ml)	Local recurrence			Local recurrence		
1	M	34	pT2 N1b	4830	75	No	No	HEP	No	No	HEP
2	M	80	pT1b N1a L1 R0	98	8	No	Yes	No	No	No	No
3	F	50	pT2 N1b L0 V0 R0	715	6	No	No	No	No	No	No
4	M	65	pT1a N1a L0 V0 R0	232	3	No	No	No	No	No	No
5	F	41	pT3b Nx	3017	18	No	No	PUL	No	No	No
6	M	49	pT3a N1b L1 V0 Pn0 R0	574	2	No	Yes	No	No	Yes	No
7	F	53	pT3 N1b L1 V1 R0	10000	181	No	Yes	HEP+OSS	No	Yes	HEP+OSS
8	M	69	pT1b N0 L0 V0 R0	4210	231	No	Yes	PUL+HEP+LYM+OSS	No	No	PUL+HEP+LYM+OSS
9	M	57	pT1b N1b L1 V1 Pn0	88	6	No	No	LYM	No	No	LYM

The patient characteristics (including age, gender, and TNM classification) of the medullary thyroid cancer patients included in this study are detailed in the table, which also presents the findings of ^68^Ga-FAPI and ^18^F-DOPA PET in identifying local recurrence, lymph node metastases, and distant metastases.

^18^F-DOPA, Fluorine-18-dihydroxyphenylalanine; ^68^Ga-FAPI, Gallium-68 fibroblast activation protein inhibitor; CEA, Carcinoembryonic antigen; F, Female; HEP, Hepatic metastases, L (L0/L1), Lymphatic invasion (L0 = no invasion, L1 = invasion), LN Mets, Lymph node metastases; M, Male; N (N0/N1/Nx), Regional lymph nodes status (N0 = no regional LN metastasis, N1, regional LN metastasis, Nx = regional LN cannot be assessed); Nr, Number (Patient identifier), OSS, Osseous metastases; PUL, Pulmonary metastases (Lung metastases); Pn (Pn0), Perineural invasion (Pn0 = no perineural invasion); R (R0), Resection margin status (R0 = complete resection with no residual tumor), T (T1/T2/T3), Primary tumor size and/or extent; TNM, Tumor, Node, Metastasis classification system, V (V0/V1), Vascular invasion (V0 = no invasion, V1 = microscopic invasion)

### ^68^Ga-FAPI versus ^18^F-DOPA: per-patient and per-lesion comparison in MCT patients

**Figure 3:**
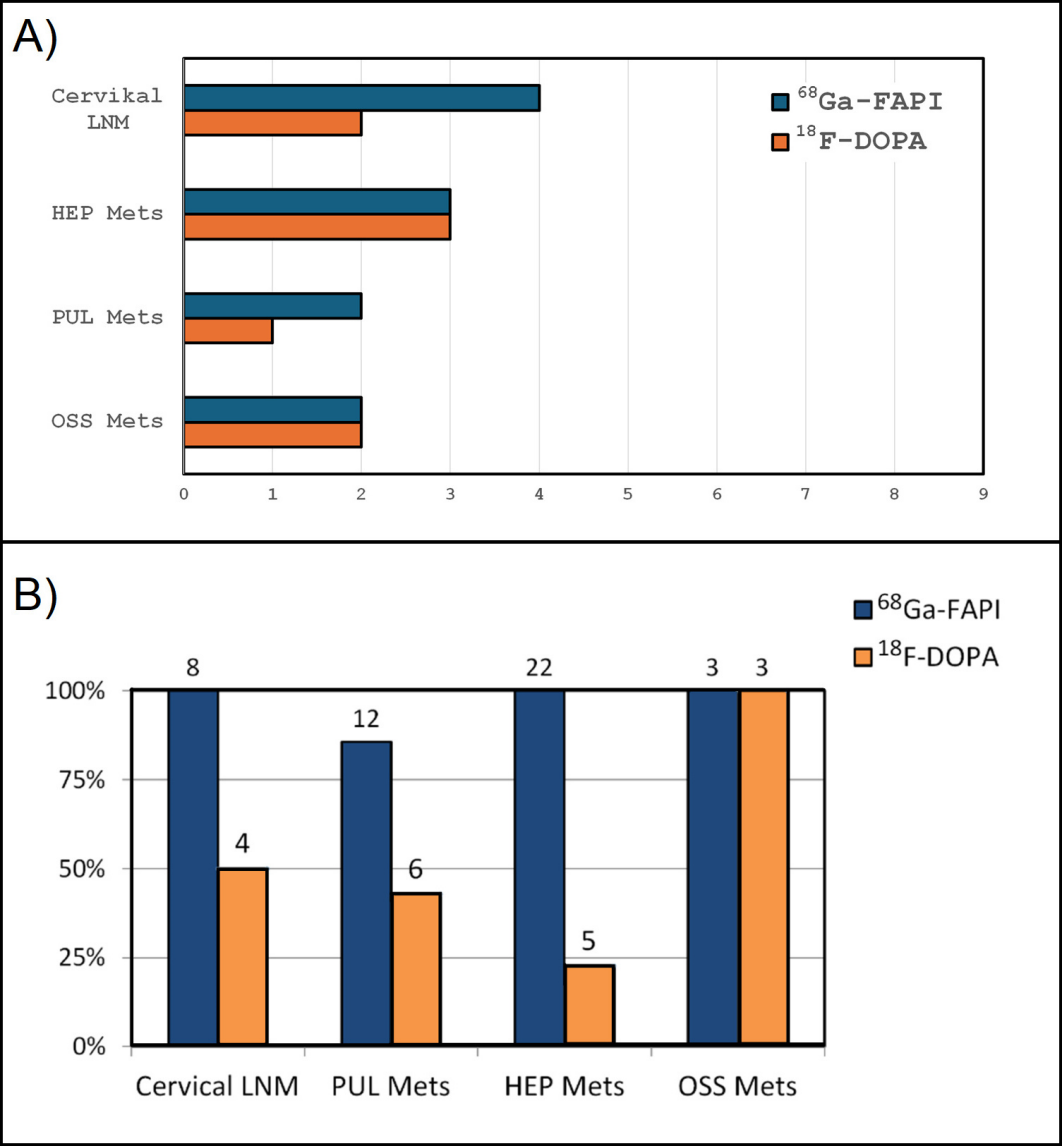
**(A)** shows the per-patient analysis results for metastases in the lymph nodes, liver, lung, and bone. **(B)** presents a per-lesion comparison of the detection rates for metastases in the lymph nodes, lung, liver, and bone, contrasting the performance of ^18^F-DOPA versus ^68^Ga-FAPI. ^68^Ga-FAPI, Gallium-68 Fibroblast Activation Protein Inhibitor; ^18^F-DOPA, Fluorine-18 Dihydroxyphenylalanine; Cervical LNM, cervical lymph node metastases; HEP Mets, hepatic (liver) metastases; PUL Mets, pulmonary (lung) metastases; OSS Mets, osseous (bone) metastases.

The findings from the per-patient and per-lesion analyses are visually summarized (**Figure 3**).

### Cervical lymph node metastases

Four of the nine patients (44%) presented with a total of eight cervical lymph node metastases (LNMs). In the per-patient analysis, ^68^Ga-FAPI detected cervical LNMs in four patients, whereas ^18^F-DOPA only depicted them in two patients. In the per-lesion analysis, ^68^Ga-FAPI PET/CT correctly identified 100% of the LNMs (true-positive [TP]: 8/8), compared to 50% with ^18^F-DOPA (TP: 4/8). The mean TBR values for true-positive LNMs were also higher with ^68^Ga-FAPI (10.9 [range: 5.6-22.8] vs. ^18^F-DOPA: 6.9 [range: 2.6-17.8]), although this difference did not reach statistical significance (p = 0.13).

### Pulmonary metastases

Three of the nine patients (33%) exhibited a total of 14 lung metastases on CT. In the per-patient analysis, more patients with lung metastases were detected by ^68^Ga-FAPI than with ^18^F-DOPA (67% (2/3) patients vs. 33% (1/3) patients). In the per-lesion analysis, ^68^Ga-FAPI correctly identified 86% more lung metastases (true-positive [TP]: 12/14) compared to ^18^F-DOPA (TP: 6/14, 43%). The mean SUVmax and TBR values of true-positive pulmonary metastases were significantly higher with ^68^Ga-FAPI (SUVmax: 4.1 [range: 1.5-8.4]; TBR: 6.1 [range: 2.2-12.5]) than with ^18^F-DOPA (SUVmax: 0.8 [range: 0.6-1.3]; TBR: 3.6 [range: 2.4-5.5]; p = 0.04 for TBR comparison).

### Bone metastases

Two of the nine patients (22%) had a total of three bone metastases. In the per-patient analysis, both ^68^Ga-FAPI and ^18^F-DOPA detected bone metastases in these two patients. On a per-lesion analysis, both modalities correctly identified all three bone metastases (100%). The SUVmax and TBR values were significantly higher with ^68^Ga-FAPI (SUVmax: 11.1 [range: 5.0-15.4]; TBR: 16.6 [range: 7.4-22.9]) compared to ^18^F-DOPA (SUVmax: 3.3 [range: 1.8-4.6]; TBR: 6.9 [range: 4.2-11.9]).

### Liver metastases

Three of the nine patients (33%) presented with a total of 22 liver metastases. In the per-patient analysis, liver metastases were detected in all three patients by both ^68^Ga-FAPI and ^18^F-DOPA. However, on a per-lesion analysis, ^68^Ga-FAPI correctly identified 100% of the liver metastases [true-positive (TP): 22/22], significantly more than ^18^F-DOPA (TP: 5/22, 44%). The SUVmax and TBR values were also significantly higher with ^68^Ga-FAPI [SUVmax: 16.2 (range: 3.5-45.6); TBR: 24.1 (range: 5.3-68.1)] compared to ^18^F-DOPA [SUVmax: 3.9 (range: 3.1-5.3); TBR: 2.7 (range: 2.1-3.7); p < 0.01 for TBR comparison].

## Discussion

This retrospective study provides preliminary insights into the comparative diagnostic performance of ^68^Ga-FAPI and the established ^18^F-DOPA-PET/CT for detecting metastases in patients with recurrent medullary thyroid carcinoma (MTC). Our findings indicate a noticeable trend toward improved lesion detection with ^68^Ga-FAPI across various metastatic sites, particularly in the lymph node, liver and lungs. This observation aligns with an expanding body of evidence suggesting the utility of FAPI-based imaging in diverse oncological entities (11, 13, 14).

In our cohort, the higher detection frequency of lymph node metastases with ^68^Ga-FAPI (100% vs. 50%) represents a crucial clinical finding. In MTC, regional lymph node involvement is a primary determinant for staging and subsequent surgical management, directly impacting prognosis (15, 16). By accurately unmasking regional lymph node disease that standard ^18^F-DOPA fails to visualize, ^68^Ga-FAPI may prevent dangerous under-staging and guide endocrine surgeons toward more tailored, complete compartmental neck dissections, potentially reducing recurrence rates. This is a critical determinant for staging and subsequent management, directly impacting prognosis and therapeutic strategies (15, 16). Enhanced identification of lesions in this crucial regional compartment may facilitate more accurate risk stratification and potentially support a more tailored approach to surgical or systemic therapy.

Similarly, the increased diagnostic yield of ^68^Ga-FAPI in identifying lung and liver metastases—common sites of distant spread in MTC (17)—highlights its capacity to improve the mapping of advanced disease. This superiority is fundamentally driven by the higher tumor-to-background ratios (TBRs) achieved with ^68^Ga-FAPI (15, 16). The higher tumor-to-background ratios (TBRs) reported with ^68^Ga-FAPI offer a plausible mechanistic hypothesis for the improved lesion visualization observed in this series. Elevated TBRs enhance image contrast, which may facilitate the identification of subtle or small metastatic deposits (18, 19). Standard ^18^F-DOPA is hampered by physiological radiopharmaceutical accumulation in organs like the liver, pancreas, and brain tissue, which can easily mask subtle metastases. ^68^Ga-FAPI, however, exhibits remarkably low physiological uptake in many normal organs. Such findings are paramount for guiding systemic treatment decisions and refining prognostic assessments, although they require validation in larger, prospective cohorts.

To evaluate these findings in a broader clinical context, the underlying cellular and structural mechanics of both imaging modalities must be considered. Standard ^18^F-DOPA targets the intrinsic metabolic activity of the neoplastic cells themselves, utilizing specific amine uptake mechanisms (20). However, its diagnostic performance faces notable constraints, particularly a diminished sensitivity in patients presenting with low or slowly rising serum calcitonin levels, or in small, indolent lesions where metabolic turnover is limited. Furthermore, physiological ^18^F-DOPA uptake in healthy tissues can occasionally obscure subtle metastatic structures. In contrast, ^68^Ga-FAPI provides an indirect yet highly effective visualization of the stromal reaction surrounding the cancer. We propose a biological rationale for these results based on the overexpression of FAP in cancer-associated fibroblasts (CAFs), a key component of the cancer stroma involved in growth and metastatic progression (21, 22). The potentially abundant presence of CAFs within the MTC microenvironment, even in lesions with lower direct cancer cell uptake of ^18^F-DOPA, could account for the enhanced detection rates and superior image contrast observed with ^68^Ga-FAPI. This highlights a clear aspect where ^68^Ga-FAPI PET/CT proves superior for clinicians: it bypasses the metabolic limitations of standard cell-targeting tracers by leveraging the robust desmoplastic stromal response characteristic of MTC, thereby providing the high lesion contrast necessary for precise disease mapping. This suggests a potential complementarity between these tracers, with ^68^Ga-FAPI demonstrating particular strength in visualizing the extent of the stromal reaction and regional tumor burden, while ^18^F-DOPA remains focused on the intrinsic amino metabolic activity of the cancer cells (8, 9). It must be noted, however, that while ^18^F-DOPA is highly specific for neuroendocrine tissue, ^68^Ga-FAPI uptake can also occur in benign inflammatory processes or tissue remodeling, which clinicians should consider during image interpretation. Future research should prioritize investigating the correlation between FAP expression levels in the cancers and ^68^Ga-FAPI uptake to better understand the biological basis of this imaging modality and to identify optimal clinical scenarios for its application.

It is important to emphasize that these results are exploratory and hypothesis-generating. The higher detection rates observed here reflect the specific characteristics of our small patient cohort (n=9) and should not be misinterpreted as definitive diagnostic sensitivity. The apparent advantage of stroma-targeted imaging over direct metabolic targeting remains a subject for further investigation, as the density of CAFs may vary between patients and disease stages. A primary limitation of this study is the absence of systematic histological confirmation as the gold standard for PET-positive findings. Instead, the assessment of diagnostic performance relied on alternative imaging modalities, specifically CT and MRI. While these are valuable tools for anatomical correlation, they lack the definitive cellular and molecular characterization offered by histopathology (1).

This reliance on cross-sectional imaging as a surrogate for histological truth introduces a potential for misclassification. For instance, findings on CT/MRI interpreted as metastases might represent inflammatory processes, leading to a possible overestimation of the true-positive rate. Conversely, small or metabolically less active metastases detectable by PET might be missed or deemed indeterminate on CT/MRI, potentially underestimating the detection capacity of either modality. This underscores the preliminary nature of the findings and highlights the need for future studies incorporating histological validation whenever feasible to definitively establish the diagnostic yield of ^68^Ga-FAPI PET/CT in comparison to ^18^F-DOPA (18, 19).

Despite these promising initial findings, the small sample size and retrospective design necessitate a cautious interpretation regarding the generalizability of the conclusions. The exclusion of three patients from the quantitative analysis due to the absence of comparative ^18^F-DOPA scans further limits the statistical power of the direct comparison. Consequently, there is a critical need for well-designed, prospective studies involving larger patient cohorts to validate these preliminary observations.

## Conclusion

In comparison, ^68^Ga-FAPI PET/CT detected a higher number of metastases with significantly higher tumor-to-background ratios compared to ^18^F-DOPA PET/CT in patients with recurrent medullary thyroid carcinoma. These preliminary findings suggest that ^68^Ga-FAPI PET/CT holds promise for improving diagnostic clarification in this challenging disease. However, these results necessitate confirmation in larger, prospective studies to definitively establish its role in the clinical management of medullary thyroid cancer.

## Data Availability

The original contributions presented in the study are included in the article/supplementary material. Further inquiries can be directed to the corresponding author.
